# Correction: YOLO-based intelligent recognition system for hidden dangers at construction sites

**DOI:** 10.1371/journal.pone.0340613

**Published:** 2026-01-06

**Authors:** The PLOS One Staff

The algorithm text appears incorrectly in the article, as it should have appeared in sequence. The correct full Algorithm are:

Algorithm 1 Pseudocode for the YOLO-CGBSE algorithm

Input: images Img, Bounding box coordinates Bx, By, width Bw, height Bℎ

Output: Class probabilities Pc and Predicted Bounding box coordinates

1: Initialize: Img = Img_train(80%) + Img_val(20%);

2: batch_size = 24;

3: epochs E = 200;

4: Generation G = 200;

5: for *i* = 1 : G do

6: for j = 1 : b*a*tcℎ_*si*z*e* do

7: Load yolo hyperparameters configuration file;

8: Run Genetic Algorithm (GA) to obtain best hyperparameter values;

9: end for

10: end for

11: Training Stage:

12: for *i* = 1 : E do

13: Load optimized yolo training parameters from GA;

14: Training on the Img_train with the YOLO-CGBSE;

15: Evaluating algorithm using Img_val;

16: end for

17: save optimal checkpoint bestweight.pt

The publisher apologizes for the errors.
