# Supplementary material for: YOLO-based intelligent recognition system for hidden dangers at construction sites
Source: PLoS One. 2025 Sep 17;20(9):e0332042. doi: 10.1371/journal.pone.0332042 (PMC12443295; doi:10.1371/journal.pone.0332042)
Supplement: Attachment — Submitted filename: Rew_Document.docx [file pone.0332042.s001.docx]

Need some improvements. Here some recommends:

1. The abstract is well-structured but can be **more concise**. Consider summarizing the contributions in fewer words to avoid redundancy.
2. Provide more in-depth explanations of the modifications made to YOLOv5n, including the specific roles and benefits of ECA, Ghost, SIOU-loss, and EIOU-NMS in enhancing detection performance.
3. Include pseudocode or algorithms to describe the implementation of the enhancements to aid in reproducibility and understanding.
4. Clearly describe the data collection process, ensuring transparency about how images were sourced, criteria for inclusion, and any preprocessing steps. Please cite this work as one of the option of working with images: Umirzakova, S., Abdullaev, M., Mardieva, S., Latipova, N., & Muksimova, S. (2024). Simplified Knowledge Distillation for Deep Neural Networks Bridging the Performance Gap with a Novel Teacher–Student Architecture. *Electronics*, *13*(22), 4530.
5. Compare the modified YOLOv5n with other state-of-the-art models in similar tasks to contextualize its performance.

Please include all this improvement in your paper
