# Supplementary material for: YOLO-based intelligent recognition system for hidden dangers at construction sites
Source: PLoS One. 2025 Sep 17;20(9):e0332042. doi: 10.1371/journal.pone.0332042 (PMC12443295; doi:10.1371/journal.pone.0332042)
Supplement: Attachment — Submitted filename: Response to Reviewers.docx [file pone.0332042.s003.docx]

**Reply to journal requirements:**

**Concern 1:** Please ensure that your manuscript meets PLOS ONE's style requirements, including those for file naming. The PLOS ONE style templates can be found at https://journals.plos.org/plosone/s/file?id=wjVg/PLOSOne_formatting_sample_main_body.pdf and https://journals.plos.org/plosone/s/file?id=ba62/PLOSOne_formatting_sample_title_authors_affiliations.pdf.

**Author response:** Thank you for your reminder. The authors have carefully reviewed PLOS ONE's formatting requirements and will ensure the manuscript fully complies with the journal's guidelines, including the file naming specifications. I have downloaded and consulted PLOS ONE's style template and will double-check all details before submission.

**Concern 2:** In your Methods section, please include additional information about your dataset and ensure that you have included a statement specifying whether the collection and analysis method complied with the terms and conditions for the source of the data.

**Author response:** Thank you for your feedback. The authors have supplemented additional information regarding the dataset in the "Method" section as requested, and have explicitly stated that the data collection and analysis methods fully comply with the terms and conditions of the data source. The relevant modifications are reflected in the “Dataset description” section of the “Method” section of the manuscript.

**Concern 3:** Please note that PLOS ONE has specific guidelines on code sharing for submissions in which author-generated code underpins the findings in the manuscript. In these cases, we expect all author-generated code to be made available without restrictions upon publication of the work. Please review our guidelines at https://journals.plos.org/plosone/s/materials-and-software-sharing#loc-sharing-code and ensure that your code is shared in a way that follows best practice and facilitates reproducibility and reuse.

**Author response:** Thank you for your reminder. The authors have carefully reviewed PLOS ONE's code sharing policy and fully understand the journal's requirements regarding the sharing of author-generated code. To ensure the reproducibility and reusability of the code, the authors have shared both the code and dataset on Figshare (DOI: https://doi.org/10.6084/m9.figshare.28644194.v1). Additionally, the generated code has been uploaded to GitHub at: https://github.com/1520133943/YOLO-based-intelligent-recognition-system-for-hidden-dangers-on-construction-sites.

**Concern 4:** We suggest you thoroughly copyedit your manuscript for language usage, spelling, and grammar. If you do not know anyone who can help you do this, you may wish to consider employing a professional scientific editing service.

The American Journal Experts (AJE) (https://www.aje.com/) is one such service that has extensive experience helping authors meet PLOS guidelines and can provide language editing, translation, manuscript formatting, and figure formatting to ensure your manuscript meets our submission guidelines. Please note that having the manuscript copyedited by AJE or any other editing services does not guarantee selection for peer review or acceptance for publication.

**Author response:** Thank you for your valuable suggestions. The authors have purchased editing services from AJE to improve the English expression of the manuscript. The editing certificate has been uploaded as an attachment to the system.

**Concern 5:** Thank you for stating the following financial disclosure:

“the Ministry of Emergency Management of the People's Republic of China，Grant numbers:Jilin-0050-2018AQ.”

Please state what role the funders took in the study. If the funders had no role, please state: "The funders had no role in study design, data collection and analysis, decision to publish, or preparation of the manuscript."

If this statement is not correct you must amend it as needed.

Please include this amended Role of Funder statement in your cover letter; we will change the online submission form on your behalf.

**Author response:** Thank you for your reminder. Regarding the role of the funder, the following statement is now declared: "The Ministry of Emergency Management of the People's Republic of China (Approval No.: Jilin-0050-2018AQ) provided funding support for this study. The funder had no role in study design, data collection and analysis, decision to publish, or preparation of the manuscript." The authors have added the above statement to the cover letter and confirmed its accuracy.

**Concern 6:** In the online submission form you indicate that your data is not available for proprietary reasons and have provided a contact point for accessing this data. Please note that your current contact point is a co-author on this manuscript. According to our Data Policy, the contact point must not be an author on the manuscript and must be an institutional contact, ideally not an individual. Please revise your data statement to a non-author institutional point of contact, such as a data access or ethics committee, and send this to us via return email. Please also include contact information for the third party organization, and please include the full citation of where the data can be found.

**Author response:** Thank you for your reminder. The authors have revised the data statement according to your request. The data and code have been publicly shared on Figshare with the DOI https://doi.org/10.6084/m9.figshare.28644194.v1, and the code has been uploaded to GitHub at https://github.com/1520133943/YOLO-based-intelligent-recognition-system-for-hidden-dangers-on-construction-sites. Therefore, the data can be accessed directly via Figshare without the need for additional contacts.

**Concern 7:** We note that Figures 9, 20, 20, 21 and 24 in your submission contain copyrighted images. All PLOS content is published under the Creative Commons Attribution License (CC BY 4.0), which means that the manuscript, images, and Supporting Information files will be freely available online, and any third party is permitted to access, download, copy, distribute, and use these materials in any way, even commercially, with proper attribution. For more information, see our copyright guidelines: http://journals.plos.org/plosone/s/licenses-and-copyright.

We require you to either (1) present written permission from the copyright holder to publish these figures specifically under the CC BY 4.0 license, or (2) remove the figures from your submission:

1. You may seek permission from the original copyright holder of Figures 9, 20, 20, 21 and 24 to publish the content specifically under the CC BY 4.0 license.

We recommend that you contact the original copyright holder with the Content Permission Form (http://journals.plos.org/plosone/s/file?id=7c09/content-permission-form.pdf) and the following text:

“I request permission for the open-access journal PLOS ONE to publish XXX under the Creative Commons Attribution License (CCAL) CC BY 4.0 (http://creativecommons.org/licenses/by/4.0/). Please be aware that this license allows unrestricted use and distribution, even commercially, by third parties. Please reply and provide explicit written permission to publish XXX under a CC BY license and complete the attached form.”

Please upload the completed Content Permission Form or other proof of granted permissions as an "Other" file with your submission.

In the figure caption of the copyrighted figure, please include the following text: “Reprinted from [ref] under a CC BY license, with permission from [name of publisher], original copyright [original copyright year].”

2. If you are unable to obtain permission from the original copyright holder to publish these figures under the CC BY 4.0 license or if the copyright holder’s requirements are incompatible with the CC BY 4.0 license, please either i) remove the figure or ii) supply a replacement figure that complies with the CC BY 4.0 license. Please check copyright information on all replacement figures and update the figure caption with source information. If applicable, please specify in the figure caption text when a figure is similar but not identical to the original image and is therefore for illustrative purposes only.

**Author response:** Thank you for your valuable comments. Figures 9, 10, 20, and 21 in the manuscript are sourced from Google and public datasets such as the SHWD dataset. Many studies, especially those related to helmet detection, have used these images, which are not subject to copyright. Therefore, the authors believe that it is unnecessary to provide written permission for their use. The individuals depicted in Figure 24 are the authors themselves, so no ethical statement is required. Overall, this study involves only the authors and does not involve any other participants.

Reply to Additional Editor Comments:

**Concern 1:** YOLO v5 was preferred for object detection operations in the study. Please explain in detail why v5 was used in particular, although it is much more up-to-date in the literature (such as YOLO v12).

**Author response:** Thank you to the editor for the valuable comments. As of now, the YOLO series algorithms have been updated to the 12th version (YOLOv12), but YOLOv5 remains the most suitable for this study due to the limited computing power of the Jetson Nano B01 used to build the system. Although later versions such as YOLOv6 to YOLOv12 offer stronger performance, they require significantly more computational resources and cannot run smoothly—or even properly—on the Jetson Nano B01 (as confirmed by updated experiments). YOLOv5 has fewer model parameters and lower computational demands, making it suitable for devices with limited performance, as demonstrated by the updated experiments. More importantly, YOLOv5 offers excellent deployment compatibility, has a very active community, and benefits from numerous tutorials, pre-trained models, and third-party tool support. Since it was released earlier, many developers have built various applications and optimization tools based on YOLOv5. This study focuses more on algorithm application rather than breakthroughs in algorithm performance, so the authors chose the more stable YOLOv5. The authors also explain this in detail in the YOLOv5 section of the Methods section of the revised manuscript.

**Concern 2:** The literature review part of the study is very limited. This part definitely needs to be detailed. In addition, a literature table consisting of columns such as "pros and cons, originality, results, used dataset" should be added.

**Author response:** Thank you for the editor’s valuable comments. In the revised manuscript, the authors have rewritten the literature review section to provide a more comprehensive overview of key literature in the relevant field. In addition, following your suggestion, we have added a comparison table of the literature (Table 1), which covers aspects such as Datasets, Method, Originality, Results, and Limitations.

**Concern 3:** Like many metrics in object detection, the choice of loss function is also very important. For this reason, please explain in detail how the two different SIOU-loss and EIOU-loss used in this study were determined and whether any customization was made at this point.

**Author response:** Thank you for the editor’s valuable suggestions. Regarding the choice of loss functions, the authors mainly referred to widely used mainstream loss functions such as EIOU-loss and SIOU-loss in existing literature. Based on this research foundation, the authors conducted systematic experimental comparisons to select the loss function most suitable for this study. It should be noted that no customized modifications were made to the loss functions in this research. The authors have added relevant explanations to the Loss function section in the Method section of the manuscript.

**Concern 4:** There are very serious deficiencies in the evaluation metrics section. For this reason, metrics such as Average Recall (AR), Optimal Localization Recall Precision (oLRP), Precision-Recall Curve and Count of predicted bounding box should also be obtained.

**Author response:** Thank you for the editor’s insightful comments regarding the evaluation metrics. The authors have carefully considered your suggestions and made the following revisions in the experimental section:

The authors have supplemented the Precision (P) curve, Recall (R) curve, PR curve, and F1 curve (see Figure 22 in the revised manuscript).

Regarding the optimal localization recall precision (oLRP) and predicted bounding box count, the authors fully understand the reviewer’s suggestions. However, due to the following considerations, these two metrics were not added in the revised manuscript:

Currently, the core evaluation metrics for YOLO series algorithm research mainly include Precision, Recall, mAP, FPS, etc. Although oLRP is a relatively new comprehensive evaluation metric, its calculation process and analysis methods have not yet been widely standardized in the current research field and are not fully compatible with the evaluation framework used in this work, which may introduce additional uncertainties. The predicted bounding box count is more suitable for multi-stage optimization or scenarios requiring precise control of the number of boxes, whereas this study mainly focuses on end-to-end detection accuracy, making this metric of limited reference value in the current setting. Recent related literature in your journal’s field has also not employed these metrics, for example:

*Research on mechanical automatic food packaging defect detection model based on improved YOLOv5 algorithm, https://doi.org/10.1371/journal.pone.0321971*

*Bearing defect detection based on the improved YOLOv5 algorithm, https://doi.org/10.1371/journal.pone.0310007.*

Since these studies focus on non-construction fields, the authors did not include them in the revised manuscript; however, their underlying algorithms are the same, so they adopt the same performance metrics. The authors ensure that the evaluation metrics used are sufficient to comprehensively reflect the performance of this method. The authors hope for your understanding.

**Concern 5:** In order to compare the results and reveal the superiority of the proposed approach in the literature, it is very important to obtain and compare the results for this dataset with several state-of-the-art models.

**Author response:** Thank you for the valuable suggestion from the editor. The authors fully agree that introducing advanced models for comparison helps to more clearly demonstrate the superiority of the proposed method. Therefore, in the revised manuscript, the authors have supplemented the Model Comparison section with several current mainstream advanced models as comparison targets, including the lightweight versions of YOLOv7 to YOLOv12, and conducted systematic experimental comparisons on the same dataset.

The relevant results are shown in Table 15, which compares the performance of each method across metrics such as Precision, Recall, F1, mAP@0.5, mAP@0.5:0.95, parameters, GFLOPs, FPS, FPS on portable devices, and model size. The experimental results demonstrate that the proposed method in this study significantly outperforms existing methods in terms of compatibility and portability, validating its effectiveness and advancement.

**Reply to review comments to the author:**

**Reply to reviewers #1:**

**Reviewer #1:** Need some improvements. Please check file. Please include all this improvement in your paper. Acknowledge the limitations of the current study, such as potential biases in the dataset or overfitting due to the model complexity.

**Author response:** Thank you for your valuable comments. The authors have carefully considered your suggestions and have made corresponding revisions and improvements to the manuscript. In the Discussion and future work section of the revised version, the authors acknowledge the current limitations of the study and propose directions for future work.

**Concern 1:** The abstract is well-structured but can be more concise. Consider summarizing the contributions in fewer words to avoid redundancy.

**Author response:** Thank you to Reviewer 1 for your recognition and suggestions regarding the abstract. The authors have carefully revised the abstract according to your comments: redundant expressions and background descriptions were removed; more concise and precise wording was used to highlight the core contributions of this paper; and the overall length of the abstract was shortened to make it clearer and more concise.

**Concern 2:** Provide more in-depth explanations of the modifications made to YOLOv5n, including the specific roles and benefits of ECA, Ghost, SIOU-loss, and EIOU-NMS in enhancing detection performance.

**Author response:** Thank you for the important comments from Reviewer 1. In the revised manuscript, the authors have provided a detailed explanation of the specific roles and performance enhancement mechanisms of ECA, Ghost, SIOU-Loss, and EIOU-NMS. The supplementary content is mainly concentrated in the Ablation Experiment section. The specific explanations are as follows:

ECA, as a lightweight channel attention mechanism, enhances feature representation, especially improving small object detection performance, while incurring minimal computational and parameter overhead. Ghost significantly reduces the number of parameters while preserving key information; it is plug-and-play with strong versatility. SIOU-loss not only optimizes the regression loss and accelerates convergence but also significantly improves detection performance in dense scenes and for small objects. EIOU-NMS more effectively removes overlapping targets, avoids false deletions, and retains more accurately localized bounding boxes, thereby improving detection accuracy.

**Concern 3:** Include pseudocode or algorithms to describe the implementation of the enhancements to aid in reproducibility and understanding.

**Author response:** Thank you for the valuable suggestion from Reviewer 1. The authors fully agree with your view. To improve the reproducibility and comprehensibility of the paper, pseudocode (Algorithm 1) has been added in the Ablation Experiment section.

**Concern 4:** Clearly describe the data collection process, ensuring transparency about how images were sourced, criteria for inclusion, and any preprocessing steps.

Please cite this work as one of the option of working with images: Umirzakova, S., Abdullaev, M., Mardieva, S., Latipova, N., & Muksimova, S. (2024). Simplified Knowledge Distillation for Deep Neural Networks Bridging the Performance Gap with a Novel Teacher–Student Architecture. Electronics, 13(22), 4530.

**Author response**: Thank you to Reviewer 1 for your careful review and constructive suggestions. The authors fully agree that providing a detailed description of the data collection and processing procedures is crucial for ensuring the transparency and reproducibility of the research. In the revised manuscript, the authors have added a Dataset Description section, which clarifies the image sources and confirms that the data collection and analysis methods fully comply with the terms and conditions of the data sources (which is also a requirement of the journal). The Dataset section has been reorganized to more clearly describe the data collection process.

However, since the referenced literature does not study the YOLO series, and the standards and methods used in that literature are not applicable to this research, the authors only referred to its structural format and therefore did not cite this literature in the revised manuscript. We hope for your understanding.

**Concern 5:** Compare the modified YOLOv5n with other state-of-the-art models in similar tasks to contextualize its performance.

**Author response**: Thank you to Reviewer 1 for the valuable suggestion. The authors fully agree that introducing advanced models for comparison helps to more clearly demonstrate the superiority of the proposed method. Therefore, in the revised manuscript, the authors have supplemented the Model Comparison section with several current mainstream advanced models, including the lightest versions of YOLOv7 to YOLOv12, and conducted systematic experimental comparisons on the same dataset.

The relevant results are shown in Table 15, which compares the performance of each method across metrics such as Precision, Recall, F1, mAP@0.5, mAP@0.5:0.95, parameters, GFLOPs, FPS, FPS on portable devices, and model size. The experimental results demonstrate that the proposed method significantly outperforms existing methods in terms of compatibility and portability, validating its effectiveness and advancement.

**Concern 6:** Please include all this improvement in your paper

**Author response:** Thank you to Reviewer 1 for the detailed review and valuable suggestions! All revisions have been incorporated into the revised manuscript, with all changes clearly marked. If the reviewer believes that any parts still require adjustment, the authors would be very happy to make further modifications. Once again, thank you for your help in improving the quality of this paper!

**Reply to reviewers #2:**

**Concern 1:** Clarity in Problem Statement

Comment: "Ensure that the problem statement is clearly defined. While the paper mentions hidden dangers, it would be helpful to specifically define what qualifies as a 'hidden danger' on construction sites (e.g., poorly stored equipment, unsafe scaffolding, electrical hazards)."

**Author response:** Thank you to Reviewer 2 for the valuable comments. The authors fully agree that clearly defining the scope of “hidden dangers” is necessary to enhance the rigor of the study. Based on your suggestion, the authors have provided a clearer definition of “hidden dangers”: these refer to potential risks on construction sites that are not easily observed directly or detected through routine inspections, including but not limited to not wearing safety helmets, smoking in no-fire zones, improper equipment storage, unsafe scaffolding, damaged cables, and so forth. The authors have added this definition in the Introduction section.

**Concern 2:** Data and Dataset Clarification

Comment: "Provide more details on the dataset used. Are the images annotated manually or are they sourced from existing databases? A brief explanation of how the data was collected and pre-processed could enhance the reader's understanding."

**Author response:** Thank you to Reviewer 2 for the valuable suggestion. The authors fully agree that providing more detailed information about the datasets used can enhance readers’ understanding. In the revised manuscript, the authors have added a Dataset Description section to clarify the sources of the images and to explain that the data collection and analysis methods fully comply with the terms and conditions of the data sources (which is also a requirement of the journal). The Dataset section has been reorganized to describe the image annotation methods and to more clearly explain the data collection process.

**Concern 3:** YOLO Implementation

Comment: "The paper mentions using YOLO for object detection. Please clarify which version of YOLO (YOLOv3, YOLOv4, YOLOv5, etc.) was used and why this version was chosen over others, especially in the context of construction site recognition."

**Author response:** Thank you to Reviewer 2 for the valuable comments. To date, the YOLO series algorithms have been updated up to version 12 (YOLOv12); however, YOLOv5 remains the most suitable for this study due to the limited computing power of the Jetson Nano B01 used in system construction. Although later versions such as YOLOv6 to YOLOv12 offer stronger performance, their higher computational demands prevent them from running smoothly—or even properly—on the Jetson Nano B01, as confirmed by updated experiments. YOLOv5 has fewer model parameters and lower computational complexity, making it well-suited for deployment on resource-constrained devices, which is also supported by our experimental results. More importantly, YOLOv5 offers excellent deployment compatibility, backed by a very active community with abundant tutorials, pretrained models, and third-party tool support. Since YOLOv5 was released earlier, many developers have created various applications and optimization tools based on it, especially for construction site recognition. This study focuses more on algorithm application rather than performance breakthroughs, so the authors chose the more stable YOLOv5. The authors have also provided a detailed explanation of this in the YOLOv5 section of the revised manuscript.

**Concern 4:** Accuracy and Performance Metrics

Comment: "The paper could benefit from more detailed performance metrics, such as precision, recall, F1 score, or mAP (mean Average Precision). A comparison of YOLO's performance versus other potential detection algorithms could provide more context for its efficacy in this application."

**Author response:** Thank you for the Reviewer 2’s suggestion. The authors have supplemented detailed performance metric analyses in the revised manuscript, including Precision, Recall, F1, mAP@0.5, mAP@0.5:0.95, parameters, GFLOPs, FPS, FPS on portable devices, and Model size. The authors ensure that these evaluation metrics comprehensively reflect the performance of the proposed method. The modifications are reflected in Tables 4 to 16 in the revised manuscript.

To further verify the effectiveness of the algorithm for this task, the authors have added several current mainstream advanced models as comparison targets in section 3.5 Model Comparison, including the lightest versions of YOLOv7 to YOLOv12, and conducted systematic experimental comparisons on the same dataset.

The relevant results are shown in Table 15, comparing the performance of each method in terms of Precision, Recall, F1, mAP@0.5, mAP@0.5:0.95, parameters, GFLOPs, FPS, FPS on portable devices, and Model size. Experimental results demonstrate that the proposed method significantly outperforms existing methods in compatibility and portability, validating its effectiveness and advancement.

**Concern 5:** Challenges and Limitations

Comment: "While the system seems promising, it would be good to address any limitations encountered during implementation. For example, how does the model handle occlusion or cluttered environments typical of construction sites? Are there any false positive or false negative challenges?"

**Author response:** Thank you sincerely to Reviewer 2 for the valuable comments! You pointed out that the model’s robustness under complex construction site environments (such as occlusion and clutter), as well as the challenges of false positives and false negatives, are indeed critical issues faced by the system. The authors take these concerns very seriously.

To address these challenges, the authors have implemented several targeted measures in this study: introducing the ECA attention mechanism to enhance feature extraction and improve detection accuracy in complex environments; adopting the EIOU-NMS algorithm to improve localization and detection of occluded targets; and employing the SIOU-loss to optimize regression performance and further enhance the overall model. These approaches effectively improve detection performance in occluded and cluttered scenarios while reducing the rates of false positives and false negatives.

However, the authors acknowledge that severe occlusion and extreme background interference remain challenges for the current system, and future work will continue to focus on improving the model’s robustness.

**Concern 6:** Real-Time Application

Comment: "The real-time application of this system is mentioned but not elaborated upon. Can the system be deployed effectively in real-world scenarios with live video feeds from construction sites? A brief discussion of the computational efficiency or hardware requirements would be valuable."

**Author response:** Thank you for Reviewer 2’s valuable comments. The authors understand the importance of deploying the system on actual construction sites; however, due to practical constraints such as safety and management restrictions on construction sites, the system has not been deployed there. Currently, the real-time deployment and validation have been completed on a school playground. Test results demonstrate that the system’s detection accuracy and speed meet practical requirements. This is detailed in the revised manuscript’s section on Deployment Model and Build System.

The system was tested on the Jetson Nano B01 platform, which has very limited computational resources. Therefore, if the system runs smoothly on this platform, it can be expected to run well on other platforms. Experiments related to computational efficiency and hardware requirements are presented in the revised manuscript’s Model Comparison section.

**Concern 7:** Safety Considerations

Comment: "It would be useful to briefly discuss how the system could be integrated with existing safety protocols on construction sites. How will the system alert workers or managers to detected hidden dangers? Are there considerations for worker privacy or data security?"

**Author response:** Thank you to Reviewer 2 for the valuable suggestion. The authors believe that integration with existing safety management platforms—such as connecting with a construction site’s BIM system to mark hazard locations in a digital twin model—is one possible way to combine the proposed system with current safety protocols. The system could also adopt a multi-level warning mechanism so that, once a potential risk is detected, alerts are sent to relevant personnel according to the grading standards of the site’s safety protocols, thereby warning workers or notifying managers.

In terms of privacy and data security, the system could employ encryption during data collection and transmission, anonymize personal identity information, and implement strict access control to comply with relevant regulations. However, each of these improvements would require a substantial workload and cooperation with construction sites. Therefore, the authors are currently unable to implement these enhancements and hope for your understanding.

These issues are also briefly discussed in the Discussion and Future Work section of the revised manuscript. The authors consider these challenges as directions for future research.

**Concern 8:** Further Improvements

Comment: "Consider adding a section on potential future work. For instance, could this system be expanded to detect additional types of hazards (e.g., environmental conditions like weather or lighting changes)? What are the next steps for improving detection accuracy?"

**Author response:** The authors sincerely thank Reviewer 2 for the valuable suggestion. Your recommendation to add a “Future Work” section and focus on system scalability (e.g., detecting more hazard types) and accuracy improvement is highly constructive, and the authors fully agree. In the revised manuscript, the authors have preliminarily outlined the future work in the Discussion and Future Work section:

First, the system will be expanded to detect additional types of hazards, such as damaged cables, and the two datasets will be merged into a single dataset to further enhance the model’s generalization capability.

Second, the detection accuracy will be further improved, especially under extreme environmental conditions, and a new module will be designed to achieve this goal.

Third, while ensuring workers’ privacy and data security, the system will be developed to integrate with existing safety protocols and functionalities, such as graded alarms and screenshot logging.

**Concern 9:** Related Work

Comment: "Please consider including a brief comparison to related work or similar AI-based hazard detection systems. How does the proposed system stand out in terms of accuracy, scalability, or real-time detection?"

**Author response:** Thank you very much for Reviewer 2’s valuable suggestion. Currently, there is no system highly relevant to this study; most existing systems only detect whether workers are wearing safety helmets. Therefore, the authors are unable to provide a system comparison as you suggested and hope for your understanding. The core of the system mainly lies in the algorithm, and the algorithm’s performance reflects the system’s overall performance. Thus, the experimental data provided in the Model Comparison section of the revised manuscript demonstrate that the proposed system achieves high accuracy and portability. In the Deployment Model and Build System section, the authors deployed the algorithm on the low-computing-power Jetson Nano B01 device and constructed the system, while YOLOv10 to YOLOv12 cannot run on this device, which further proves the system’s compatibility and scalability.

**Concern 10:** Conclusion

Comment: "The conclusion should not only summarize the findings but also emphasize the practical implications of the system for improving construction site safety. A brief mention of its potential impact on reducing accidents or improving safety audits could strengthen the conclusion."

**Author response:** Thank you to Reviewer 2 for the valuable suggestion. In the revised manuscript’s Conclusion section, the authors have added content regarding the practical impact of the system on improving construction site safety. This addition allows the conclusion to more comprehensively reflect the research findings and their real-world significance.
